# A Soft Voting Ensemble-Based Model for the Early Prediction of Idiopathic Pulmonary Fibrosis (IPF) Disease Severity in Lungs Disease Patients

**DOI:** 10.3390/life11101092

**Published:** 2021-10-15

**Authors:** Sikandar Ali, Ali Hussain, Satyabrata Aich, Moo Suk Park, Man Pyo Chung, Sung Hwan Jeong, Jin Woo Song, Jae Ha Lee, Hee Cheol Kim

**Affiliations:** 1 Institute of Digital Anti-Aging Healthcare, Inje University, Gimhae 50834, Korea; sikandarali@live.inje.ac.kr (S.A.); alihussain4541@live.inje.ac.kr (A.H.); 2 Department of Computer Engineering, Institute of Digital Anti-Aging Healthcare, Inje University, Gimhae 50834, Korea; jamessatya@inje.ac.kr; 3 Department of Internal Medicine, Division of Pulmonology, Institute of Chest Diseases, Severance Hospital, Yonsei University College of Medicine, Seoul 03722l, Korea; PMS70@yuhs.ac; 4 Samsung Medical Center, Division of Pulmonology and Critical Care Medicine, Sungkyunkwan University School of Medicine, 81, Irwon-ro, Gangnam-gu, Seoul 06351, Korea; mpchung@skku.edu; 5 Gil Medical Center, Department of Internal Medicine, Gachon Medical School, Incheon 21565, Korea; jsw@gilhospital.com; 6 Division of Pulmonology and Critical Care Medicine, University of Ulsan College of Medicine, Asan Medical Center, Seoul 05505, Korea; jwsong@amc.seoul.kr; 7 Department of Internal Medicine, Division of Pulmonology, Inje University of College of Medicine, Haeundae Paik Hospital, Busan 48108, Korea; 8 College of AI Convergence, Institute of Digital Anti-Aging Healthcare, u-AHRC, Inje University, Gimhae 50834, Korea

**Keywords:** idiopathic pulmonary fibrosis disease, machine learning, soft voting ensemble, machine learning prediction

## Abstract

Idiopathic pulmonary fibrosis, which is one of the lung diseases, is quite rare but fatal in nature. The disease is progressive, and detection of severity takes a long time as well as being quite tedious. With the advent of intelligent machine learning techniques, and also the effectiveness of these techniques, it was possible to detect many lung diseases. So, in this paper, we have proposed a model that could be able to detect the severity of IPF at the early stage so that fatal situations can be controlled. For the development of this model, we used the IPF dataset of the Korean interstitial lung disease cohort data. First, we preprocessed the data while applying different preprocessing techniques and selected 26 highly relevant features from a total of 502 features for 2424 subjects. Second, we split the data into 80% training and 20% testing sets and applied oversampling on the training dataset. Third, we trained three state-of-the-art machine learning models and combined the results to develop a new soft voting ensemble-based model for the prediction of severity of IPF disease in patients with this chronic lung disease. Hyperparameter tuning was also performed to get the optimal performance of the model. Fourth, the performance of the proposed model was evaluated by calculating the accuracy, AUC, confusion matrix, precision, recall, and F1-score. Lastly, our proposed soft voting ensemble-based model achieved the accuracy of 0.7100, precision 0.6400, recall 0.7100, and F1-scores 0.6600. This proposed model will help the doctors, IPF patients, and physicians to diagnose the severity of the IPF disease in its early stages and assist them to take proactive measures to overcome this disease by enabling the doctors to take necessary decisions pertaining to the treatment of IPF disease.

## 1. Introduction

Idiopathic pulmonary fibrosis (IPF) is one of the most deleterious interstitial fibrosing diseases. It is a chronic disease with unknown etiology and it is very difficult to diagnose its actual cause. It is a typical progressive interstitial lung disease condition in which scars occur in the lungs of the patient. It has been classified and considered as a form of idiopathic interstitial pneumonia. It is mostly prominent in older adults without any known cause with worsening conditions of cough and dyspnea [1]. The average survival time of this disease is very alarming. The patients could hardly survive for 2~5 years after the initial diagnosis of this disease [2]. It affects the performance of the lungs unpredictably and as a result, the patient feels difficulty in breathing, and it disturbs the whole respiratory system [3]. It exacerbates the lung condition and its performance and at the same time causes distortion to the shape and architecture of the pulmonary system. This leads to hypoxia in patients, which is a disease condition in which the body of the patient does not get adequate oxygen supply. Sometimes it causes failure in the whole respiratory system and causes death [4]. IFP has certain characteristics like the abnormal proliferation of alveolar epithelial cells, the proliferation of myofibroblasts, and the excessive accumulation of extracellular matrix [5]. As IPF is a very complex kind of disease, currently, there is no proven treatment or therapy for this disease [6]. Researchers have been trying different approaches to find the factors which cause this disease and they have been applying different techniques for the early diagnosis of this disease as well [7,8]. Although many research studies have been conducted for the diagnosis of IPF, there is still enough room for improvement. Artificial intelligence has opened new avenues in the healthcare sector as well, as it has revolutionized other domains. Nowadays researchers have been using machine learning, deep learning, and other artificial intelligence techniques for the diagnosis of all kinds of diseases including lung disease [9,10,11]. 

Research Motivation and Research Goal

As IPF is a very complicated chronic disease with unknown reasons and its early diagnosis and prognosis have been a challenging task for physicians and doctors [12,13,14]. The traditional ways of diagnosing this disease are based on statistical methods like the regression model or cox proportional hazard model [15,16] and they are not data-driven. They are very time-consuming, have low accuracy, and are less effective in addressing this lethal disease. With the advent of artificial intelligence technology, new avenues and opportunities have been created for understanding and interpreting IPF disease and its different severity levels. In this research study, we aim at applying the latest artificial intelligence techniques like machine learning approaches for the early diagnosis of exacerbations of IPF disease which will assist the physicians and doctors to diagnose this disease in a reliable and efficient way. It will help to choose the best treatment strategies for the IPF patients by avoiding the deterioration of disease in its early stages. Contrary to the traditional models which are based on statistical methods, this study will investigate the machine learning-based data-driven models. Such models are more reliable and accurate. This research study will also help expedite the process of diagnosing this disease with high accuracy.

The main contribution of this paper:

◦ Applying different state-of-art machine learning algorithms on IPF data;
◦ Investigating the performance of all applied machine learning algorithms;
◦ Developing a soft voting ensemble model for the prediction of severity of IPF disease in patients with lung disease;
◦ Evaluating the performance of the proposed soft-voting ensemble-based model; and
◦ To the best of our knowledge, we are among the pioneers for applying machine learning and proposing a soft-voting ensemble approach on an IPF dataset with improved accuracy.

The rest of this research paper is organized as follows. Related research work has been discussed in Section 2. Section 3 elucidates the material and methods used in this research for the early prediction of IPF disease severity. Section 4 contains the details pertaining to our proposed soft voting ensemble approach. The experimental results are described and discussed in Section 5. Conclusion and research limitations have been shown in Section 6 of this research paper.

## 2. Related Works

Many research studies have been done to figure out solutions for idiopathic pulmonary fibrosis disease. In the past, researchers used traditional statistical methods for the prediction, classification and, diagnosis [17] of this disease but nowadays the latest technology has been used for this purpose. Yu Shi et al. [18] used quantum particle swarm optimization, which is a random forest approach for the prediction of idiopathic fibrosis progression. They used a dataset of features having the region of interest (ROI) obtained from high-resolution computed tomography. In order to extract the important features, they applied the wrapper method. It combined quantum particle swarm optimization with the well-known machine learning classifier random forest. They applied their model on 50 IPF patients’ datasets which were taken from different medical centers. The overall accuracy of their model was 82.1%. They also calculated the other performance measures like the sensitivity and specificity of their model. Simon L F Walsh MD et al. [11] applied deep learning on high-resolution CT images for the classification of fibrotic lung disease. They used 1157 high-resolution CT scans for their experiment, which were taken from two institutions. They divided the dataset into three cohorts. They resampled the scans and converted them into image montages. They got 420,096 unique montages and this dataset was used for training the algorithm. The model performance was evaluated with the accuracy, weighted k coefficients, and prognostic accuracy. They evaluated the model with test set A having 139 data samples and test set B with 150 high-resolution CT scans. The model showed an accuracy of 76.4% for test set A. The median accuracy of the model for test set B was 70.7%. Christe, Andreas MD et al. [19] applied deep learning for the diagnosis of pulmonary fibrosis. They tried to find out the performance of a computer-aided diagnosis system as compared to the human reader. They used 105 pulmonary fibrosis patients’ data. They found that the classification accuracy of computer-based machine learning algorithms was similar to those of human readers for the classification of IPF disease. Ali Hussain et al. [20] used machine learning techniques for the forecasting of exacerbation in patients having chronic obstructive pulmonary (COPD) disease. They used five base classifiers and developed an ensemble classifier for the prediction of COPD. They used 24 attributes for their experiment. They calculated the accuracy, precision, recall, f measure, and AUC of all the machine learning classifiers which they have used in their experiment. Their proposed ensemble model showed an accuracy of 91.0849%, an AUC score of 96.8656%, a precision of 90.7725%, a recall of 91.3607%, and an F-measure of 91.0656%, respectively. Sang Cheol Park et al. [21] also used a computer-aided system for the detection of interstitial lung disease. They applied Artificial neural networks (ANN) on CT images by selecting the optimal features with the help of an optimization method using a genetic algorithm. They trained the ANN model by the back-propagation method and minimized the total square error of the model. This computer-aided detection system showed the performance results with 80.0% sensitivity, 85.7% specificity, and area under the receiver operating characteristic curve (AUC = 0.884), respectively. 

## 3. Research Materials and Methods

This section describes the experimental and research methods of this research study thoroughly.

### 3.1. Data Source

To conduct the experiment for this research study, we used data of patients from the Korea interstitial lung disease cohort (KICO) data. This study was approved by the institutional review board with IRB No. 2021-07-017 for Haeundae Paik Hospital, Busan, South Korea, and the requirement for written informed consent was waived due to the retrospective nature of this study. This dataset has 2424 IPF patients. It contains all the necessary information about the patients. It contains 502 different kinds of features related to the patients.

This dataset contains information about the demography of the patient, drug information, medical history of the patient, drug information, survival, death and exacerbation information, and different pulmonary function information, etc.

IPF disease prediction can be made on different research approaches and methodologies. In the past, researchers used the traditional approaches for its prediction and preprocessed the data accordingly. We are using the latest AI approaches for its prediction, and we preprocessed the data for IPF patients having an acute exacerbation, pneumonia, some other infections like sepsis, pulmonary embolism, lung cancer, heart disease, worsening of IPF patients’ conditions due to some other disease and lastly the death of the patient due to IPF disease. The data have been labeled into four different exacerbation stages of the patients based on the number of occurrences of exacerbation i.e., class 0 represents mild IPF disease condition with no exacerbation occurrences. Class 1 represents the exacerbation state of the IPF patient at the first stage. The patients who are falling in this category have experienced the occurrence of an exacerbation one time during the follow-up. Class 2 shows exacerbation at the second stage because these patients have experienced exacerbation two times, and class 3 represents the severe exacerbation of the IPF patients because they underwent three or more exacerbations. We investigate and predict the patients’ disease condition based on these aforementioned conditions.

### 3.2. Data Preprocessing

Data preprocessing is one of the most important parts of AI application. The performance of the AI model and the predictive behavior is highly dependent on data preprocessing [22]. It is a process in which the raw data are cleaned, transformed, integrated, normalized, reduced, and eventually made fit for the training of any kind of machine learning or deep learning model by using different kinds of techniques [23]. The data may contain missing values, inconsistent data, noisy data, null values, and other kinds of redundancies. We need to remove these kinds of outliers from the dataset, and it can be done by the data cleaning approach. Other than that, we need to perform data transformation, data integration, data normalization, and data reduction.

We performed data preprocessing for our dataset and applied different approaches to making the data fit for the model implementation. The first-hand dataset was a raw dataset that contained so many inconsistencies and it was a noisy dataset. It contained missing values and incorrect data. We identified those discrepancies in our dataset and removed them. We discarded the data of patients for whom more than 70% of the data were missing and the rest of them were replaced with mean values by using the k-nearest neighbor imputation method [24,25]. Some of the data were incorrectly placed, i.e., there was mismatching in data types or some special characters. There was also some unnecessary information that was also removed from the dataset like doctors’ visits, center name, chart number, etc. We use one-hot encoding for the categorical data. For example, to convert the gender into binary values we used the one-hot encoding technique and assigned male as 1 and female as 0. Likewise, most of the features had categorical values Yes as 1 and No as 0. In order to find the highly relevant features, we used the tree-based ensemble approach, which is a well-known feature selection technique [26].

### 3.3. Data Sampling

Data sampling is a technique which is used for balancing imbalanced data [27]. In most cases, the data of each class in the target labels are not equal. Some classes have a higher number of data and others have a low amount of data. This condition is called data imbalance. With the imbalanced data, the model is vulnerable to overfitting. In most cases the model’s behavior is biased, and it does not give an actual performance. Data sampling is used to overcome this issue. There are mainly two kinds of data sampling, i.e., oversampling and under-sampling. In data oversampling the data of the minority class are enhanced and equalized to the majority class. In data under-sampling the majority class data are reduced and equalized to the minority class, however, this technique causes the loss of important data. In our experiment, we have used the Synthetic minority oversampling technique (SMOTE) [28] which is one of the most popular data oversampling approaches. SMOTE selects the instance of minority class randomly and searches its k nearest minority class neighbors. Then, while choosing one of the k nearest neighbors, synthetic instances are generated randomly. In this way, it synthetically creates new data samples for the minority classes, and we applied this method to our dataset to deal with the imbalanced data. It improves the learning of our model by reducing the bias introduced due to the unbalancing of the class.

### 3.4. Feature Selection

Feature selection plays a vital role in the optimal performance of the model. It is a process of finding useful features from a huge amount of data. It filters out irrelevant and redundant features from the dataset. It helps to select the best features from the high-dimensional data. There are three main feature selection methods, namely filters, wrappers, and the embedded/hybrid method. In our experiment, we used the recursive feature elimination method (RFE) for the selection of highly relevant features from the high-dimensional data [29]. Considering the expert opinion from the doctors, 26 highly relevant features were chosen from the dataset. The features are gender, height (ht), weight (wt), body mass index (BMI), age, job, toxic chemical, toxic wood dust, toxic mineral dust, smoking, diagnosis age, bronchoalveolar lavage (BAL), arterial blood gases (ABG), pulmonary function test forced expiratory volume measure (PFT_FEVm), pulmonary function test forced expiratory predicted value (PFT_FEVpc), pulmonary function test forced vital capacity (PFT_FVCm), pulmonary function test forced vital capacity predicted value (PFT_FVCpc), pulmonary function test free fluid (PFT_FFpc), diffusing capacity of lungs for carbon monoxide measure (PFT_DLCOm), diffusing capacity of lungs for carbon monoxide predicted value (PFT_DLCOpc), ground-glass opacity (GGO), rhema, anti-CCP, NT-Pro BNP, echo, home oxygen treatment. Table 1 shows all the features used in this research work.

## 4. Proposed Ensemble Model

For the development of our proposed model, we opted three high-performing machine learning classifiers, namely gradient boosting (GB), random forest (RF), and extra gradient boosting (XGB). We developed our soft voting ensemble model based on these three basic classifiers. The performance measures of these algorithms were comparatively higher than the other machine learning algorithms, and these classifiers showed the best prediction result for early prediction and diagnosis of severity of IPF disease patients. We chose the soft voting ensemble (SVE) approach in which the different exacerbations stages of the IPF disease were classified based on the probabilities of all the predictions made by the base classifiers. The proposed SVE model takes the class which has maximum probability and sums up all the probabilities of each class predicted by each base classifier. Lastly, our proposed SVE model predicts the class which has a high probability value.

(1) $$\mathrm{SVE}=\;\frac{1}{N}Max\sum \;P_{\mathrm{GB}}\;+\;P_{\mathrm{RF}}\;+\;P_{\mathrm{XGB}}$$

where “*N*” represents the number of base classifiers, and “P” denotes the probability of base classifiers. The pseudo-code of our research work has been presented in Algorithm 1.
**Algorithm 1** To Develop a Soft Voting Ensemble Model for the Prediction of IPF Disease Severity1Let’s the whole IPF dataset consists of i instances and X features.2The class variable is Y so labels_Yi = [4]3Function F: **X**→ labels_Yi4**Procedure** KNN Imputation (IPF_dataset)5 **Procedure** Split_data (IPF_dataset)6  Training_data, Testing_data = split (IPF_dataset)7**Procedure** datasampling (IPF_dataset)8  return (IPF_dataset)10C1 = GBM (Training_dataset, Testing_data)12C2 = RF (Training_dataset, Testing_data)13C3 = XGB (Training_dataset, Testing_data)14**Procedure** ensemble_model (Training_dataset, Testing_data)15  soft_voting_ensemble = concatenate (C1, C2, C3)16  soft_voting_ensemble.fit (Training_dataset, Testing_data)17  Predictions = soft_voting_ensemble.predict (Testing_data)

### 4.1. Why Soft Voting Ensemble Is Better and Conspicuous among Other Classifiers

Ensemble methods are a very popular approach in the domain of artificial intelligence. These methods use multiple kinds of machine learning models and combine the results of all the models and give better performance. Initially, all the base classifiers are trained, and every classifier gives the individual decision. These individual decisions are combined based on the probability values specifying the predicted value to a particular class. Customized weights are also assigned to the specific class in the base classifiers which have a higher importance in the prediction and classification process. The soft voting ensemble uses the aggregation of mean and weighted majority voting phenomena which selects the greatest probability value for the target label. It helps to balance out the weaknesses of individual classifiers. Ensemble methods are used, aiming to reduce the bias and variance of individual classifiers. It also provides parameter diversity of different classifiers enabling one to choose the best parameters from the base classifiers. Furthermore, it offers structural diversity and multi-objective optimization which is also an amazing breakthrough in this approach [30].

### 4.2. Overall Workflow of the Proposed Ensemble Model

**Figure 1:**
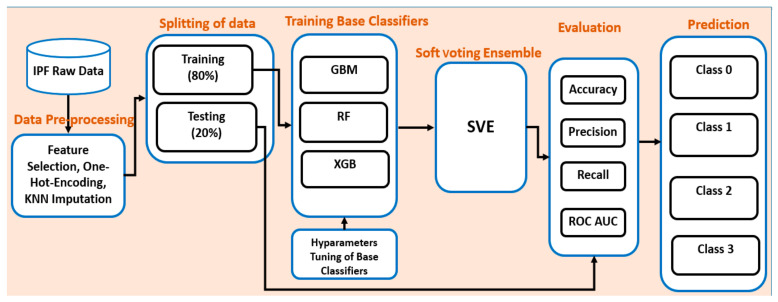
Overall workflow of proposed soft voting ensemble model.

We developed our proposed ensemble model by integrating three different state-of-the-art machine learning classifiers. The overall workflow of the proposed research study has been shown in Figure 1. First, we extracted the data from the KICO dataset by removing the redundant data from it. The dataset consisted of so many inconsistencies and irrelevant information which were not in line with our target research goal. We needed to identify all those irrelevant data and discard them from the cohort of our dataset. Second, the most relevant features have been selected from the high dimensional dataset using the recursive feature elimination (RFE) technique. Third, the dataset has been divided into training and testing datasets, i.e., 80% for training the model and 20% for the testing of the model. Fourth, as our data were imbalanced, we needed to balance it so that we can get the optimal performance of the model without any model overfitting. To address this problem, we applied the data oversampling technique (SMOTE) [28] which makes the data balanced for the implementation of models, and then all the five base classifiers were trained. We applied hyperparameter tunning to get the optimal result of the base classifiers. Fifth, the results of all the base classifiers were combined to develop our proposed ensemble-based model. Sixth, the proposed ensemble model was evaluated by calculating the performance measures such as accuracy, precision, recall, and F1-score. Lastly, the model predicts all the four stages of IPF exacerbation occurrences and makes classification as its output.

### 4.3. Implementation Environment

All these experiments and analysis of data were performed using a 64-bit Windows operating system with Intel(R) Core (TM) i7-7700 CPU @ 2.60 GHz, 3.60 GHz processor and 8 GB installed RAM. Anaconda version 4.9.2 (Anaconda Inc., Austin, TX, USA) framework was used. Jupyter Notebook, an open-source web application, has been used for the implementation and development of the models by using Python language (Version 3.6.8, Python software foundation, Wilmington, DE, USA), Keras (Version 2.4.3, MIT, Cambridge, MA, USA, and Scikit-learn libraries.

## 5. Experimental Results and Discussion

This section elucidates the findings of our experiment. We have shown the experimental results like accuracy, AUC, precision, recall and, F-1 score of all the applied machine learning algorithms. The ROC curves of the machine learning algorithm have also been shown in this section.

### 5.1. Experimental Results

We conducted our experiment on different base classifiers. We got the accuracy for each classifier and combined the results to develop a voting ensemble classifier which cumulatively gives a good result as compared to the individual classifier. We calculated different performance measures like accuracy, precision, and recall, etc., of our proposed model. These results were compared with the results of other traditional machine learning models. From the experimental result, we found that our proposed model outperformed the rest of the compared models. The results of the models have been shown in Table 2.

### 5.2. Evaluation

**Figure 2:**
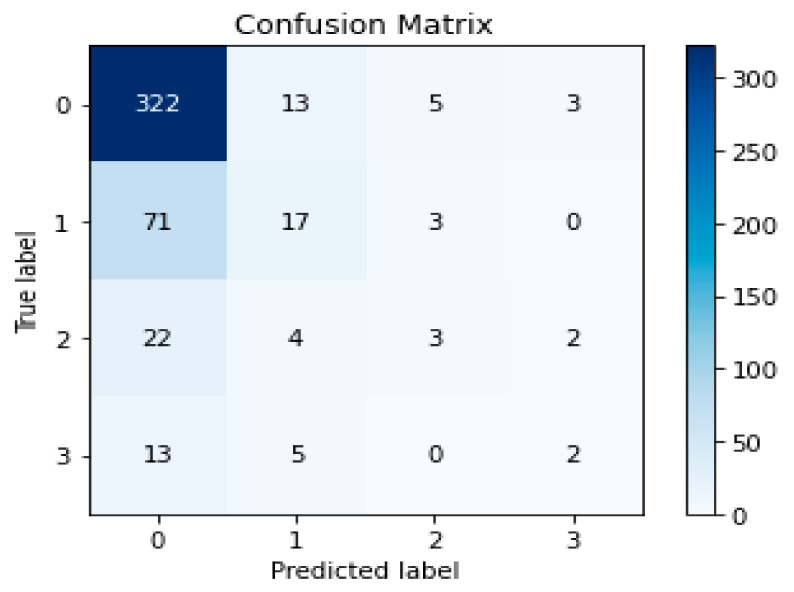
Confusion matrix for IPF disease prediction.

Basically, a confusion matrix represents a tabular summary of the model. It shows the number of correctly and incorrectly predicted values by the classifier. It provides better intuition pertaining to the performance of the model. It has two dimensions, one dimension represents the actual classes, the other dimension shows the classes predicted by the model. The confusion matrix of the proposed model has been shown in Figure 2. On the *y*-axis, it represents the actual class labels from 0 to 3 which denote mild IPF disease, IPF exacerbation stage 1, IPF exacerbation stage 2 and, IPF exacerbation stage 3, accordingly. Furthermore, the predicted labels have been shown on the *x*-axis of the confusion matrix table. The values in the diagonal showed the IPF disease prediction rate of each class. Moreover, the proposed soft-voting ensemble class had a satisfactory IPF disease prediction.

**Figure 3:**
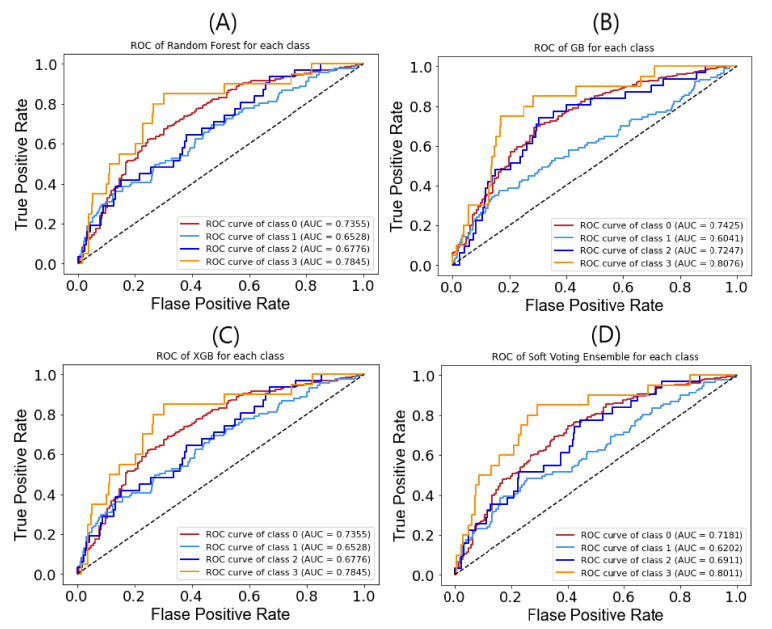
ROC of all the base classifiers and our proposed soft voting ensemble classifier for each class. (**A**). ROC of RF; (**B**) ROC of GB; (**C**) ROC of XGB; (**D**) ROC of proposed SVE. Note: Class 0 denotes mild IPF; class 1 IPF exacerbation stage 1; class 2 IPF exacerbation stage 2; IPF exacerbation stage 3.

The receiver operating characteristic (ROC) curve is also one of the criteria to examine the performance of the model. It shows the reliability of the model through graphical representation. The ROC curve gives the graphical illustration of the trade-off between the true positive and true negative. The more the graph is closer to the top left corner, the higher its performance and discriminatory ability of the model. The true positive rate which is also known as sensitivity is indexed on the *y*-axis and the false positive rate is indexed along the *x*-axis. In our research study, we also evaluated the performance of all the base classifiers and our proposed soft voting ensemble model by finding out the ROC curves. These curves have been used to evaluate and determine the diagnostic performance of our proposed ensemble model for the IPF disease. The ROC curves of all the base classifiers and our proposed ensemble model have been shown in Figure 3. The AUC of our proposed soft voting ensemble for class 0, class 1, class 2, and class 3 are 0.7181, 0.6202, 0.6911, and 0.8011, respectively.

### 5.3. Discussion

The aim of this research study was to apply machine learning techniques for the prediction of different severity stages of IPF disease patients. The patients were categorized based on the occurrences of exacerbation and their subsequent hospital visits for the treatment. Multiclass classification of IPF disease was performed by using different machine learning models. We applied several state-of-the-art machine learning algorithms on our dataset, Korea interstitial lung disease cohort (KICO) from Haeundae Paik Hospital. We applied several machine learning models to our dataset. Some of the models gave very good results; however, the performance of some applied machine learning algorithms was not so good. We chose best performing machine learning models from the pool of machine learning models which were applied to our dataset. The best performing classifiers were gradient boosting (GB), random forest (RF), and extra gradient boosting (XGB). Considering these base classifiers, we developed an ensemble-based model which gave a better performance as compared to the individual classifier. There are two ensemble approaches, i.e., soft voting and hard voting ensemble, and we applied both approaches. We analyzed and compared the results of both approaches. The results revealed that a soft voting ensemble is best for our dataset. The experimental results have been shown in Table 2. Different performance measures, for example, accuracy, precision, recall, and f-1 score, of each base classifier and the proposed ensemble model, were calculated. The accuracy, precision, recall, and f1-score values for GBM were (0.6907, 0.6300, 0.6900, 0.6400), (0.6948, 0.6300, 0.6900, 0.6600) for random forest, (0.6989, 0.6300, 0.7000, 0.6400) for extra gradient boosting, and, (0.7100, 0.6400, 0.7100, 0.6600) for our proposed soft voting ensemble model. From the experimental findings, it is concluded that our proposed ensemble-based model outperformed all the other machine learning models. Furthermore, our ensemble approached showed good performance with optimal output among the applied machine learning models in the early prediction and classification of IPF disease patients.

### 5.4. Limitations

There are some limitations to this research study. Machine learning models require a plethora of data to be used for the training of the model, however in our case, we did not have enough data for the training of the model which could foster a high result. Furthermore, the quality of our data was not good and was very inconsistent, and contained null values which also rendered a low accuracy of the models, but the collective accuracy of our proposed SVE model is higher than the base classifiers. In our future research, we are aiming to collect more data with good quality and try to enhance the accuracy of the models.

### 5.5. Internal and External Thread to the Validity

We also considered the potential threats to the validity of this study. One of the threats associated with the internal validity of the model is overfitting. To address this threat, we applied 5-fold cross-validation which ensured the validity of our proposed model. The external validity of our model pertains to the generalization of our model. We have trained our model with a diversity of data which also contained a mixture of imbalanced data. If the model is used for any other closely related purposes it will retain its performance in such circumstances as well.

## 6. Conclusions and Future Work

This research study proposed a soft voting ensemble-based model for the early prediction and classification of the idiopathic pulmonary fibrosis severity in patients with lung disease. For the development of this proposed ensemble model, we used the KICO dataset. This IPF dataset contained 2424 patients’ data with 502 different attributes. The dataset was preprocessed by applying different data cleaning and preprocessing techniques. The missing values were filled by the K-nearest neighbor imputation technique. Highly relevant features were selected by the recursive feature elimination technique. The dataset was imbalanced, therefore, SMOTE, an oversampling technique, was used to overcome the data imbalance issue. We applied multiple machine learning models. From the pool of machine learning models, only the three best performing state-of-the-art machine learning models (gradient boosting machine, and extreme gradient boosting) have been used as base classifiers. All the base classifiers were trained and evaluated by conducting hyperparameter tunning. The optimal hyperparameters were selected for the training of the base classifiers. The prediction results of all the base classifiers were combined to construct our proposed model. The accuracy of our proposed SVE model was 71.00%, precision 64.00, recall 71.00, and f-measure 66.00. Consequently, our proposed SVE model outperformed all the applied machine learning models with optimal prediction and classification accuracy. This proposed SVE model will surely help alleviate and mitigate the IPF disease severity and deterioration in its early stages. It will also ensure effective treatment by beefing up the treatment procedures and reduce the rate of mortality and morbidity of IPF patients.

In the future, we aim to collect more data of IPF disease patients and apply other advanced machine learning techniques particularly deep learning techniques which demonstrate high accuracy with a good amount of data. The more we have data, the better the accuracy, so we will try to enhance the prediction accuracy of IPF patients with more data.

## Figures and Tables

**Table 1:** Feature’s information.

	Features	Feature Details	Feature Type
1	Sex	Male = 1, female = 0	Categorical
2	Ht	Height	Continuous
3	Wt	Weight	Continuous
4	BMI	Body mass index	Continuous
5	Age	Age of patient	Continuous
6	Job	Housewife = 1, Office worker = 2, commerce = 3, construction site = 4	Categorical
7	Toxic_chem	Toxic chemical yes = 1, No = 0	Categorical
8	Toxic_wooddust	Toxic wood dust yes = 1, No = 0	Categorical
9	Toxic_mineraldust	Yes = 1, No = 0	Categorical
10	Smoking	Yes = 1, No = 0	Categorical
11	DxAge	Diagnose age	Continuous
12	BAL_Done	Bronchoalveolar lavage, yes = 1, No = 0	Categorical
13	ABG_Done	Arterial blood gases, yes = 1, No = 0	Categorical
14	PFT_FEVm	Pulmonary function test forced expiratory volume measure	Continuous
15	PFT_FEVpc	Pulmonary function test forced expiratory predicted value	Continuous
16	PFT_FVCm	Pulmonary function test forced vital capacity	Continuous
17	PFT_FVCpc	Pulmonary function test forced vital capacity predicted value	Continuous
18	PFT_FFpc	Pulmonary function test free fluid	Continuous
19	PFT_DLCOm	Diffusing capacity of lungs for carbon monoxide measure	Continuous
20	PFT_DLCOpc	Diffusing capacity of lungs for carbon monoxide predicted value	Continuous
21	CT_GGO	Ground-glass opacity, Yes = 1, No = 0	Categorical
22	RheumaYN	Rhema, Yes = 1, No = 0	Categorical
23	AntiCCPYN	Anti-CCP, Yes = 1, No = 0	Categorical
24	NT-Pro BNP	NT-Pro BNP, Yes = 1, No = 0	Categorical
25	EchoYN	Echo, Yes = 1, No = 0	Categorical
26	RxHomeYN	Home oxygen treatment, Yes = 1, No = 0	Categorical

**Table 2:** Comparison with other machine learning models.

Classifiers	Accuracy	Precision	Recall	F1-Score
GBM	0.6907	0.6300	0.6900	0.6400
RF	0.6948	0.6300	0.6900	0.6600
XGB	0.6989	0.6300	0.7000	0.6400
**SVE**	**0.7100**	**0.6400**	**0.7100**	**0.6600**

Note: SVE denotes the proposed soft voting ensemble shown in bold; GBM gradient boosting machine; RF Random Forest; XGB extra gradient boosting.

## References

[1] Raghu G., Collard H.R., Egan J.J., Martinez F.J., Behr J., Brown K.K., Colby T.V., Cordier J.F., Flaherty K.R., Lasky J.A. (2011). An official ATS/ERS/JRS/ALAT statement: Idiopathic pulmonary fibrosis: Evidence-based guidelines for diagnosis and management. Am. J. Respir. Crit. Care Med..

[2] Raghu G., Freudenberger T.D., Yang S., Curtis J.R., Spada C., Hayes J., Sillery J.K., Pope C.E., Pellegrini C.A. (2006). High prevalence of abnormal acid gastro-oesophageal reflux in idiopathic pulmonary fibrosis. Eur. Respir. J..

[3] Idiopathic Pulmonary Fibrosis (IPF). https://www.webmd.com/lung/what-is-idiopathic-pulmonary-fibrosis.

[4] Kim S.Y., Diggans J., Pankratz D., Huang J., Pagan M., Sindy N., Tom E., Anderson J., Choi Y., Lynch D.A. (2015). Classification of usual interstitial pneumonia in patients with interstitial lung disease: Assessment of a machine learning approach using high-dimensional transcriptional data. Lancet Respir. Med..

[5] Wolters P.J., Blackwell T.S., Eickelberg O., Loyd J., Kaminski N., Jenkins G., Maher T.M., Molina M.M., Noble P.W., Raghu G. (2018). Time for a change: Is idiopathic pulmonary fibrosis still idiopathic and only fibrotic?. Lancet Respir. Med..

[6] Selman M., Pardo A. (2001). Idiopathic pulmonary fibrosis: An epithelial/fibroblastic cross-talk disorder. Respir. Res..

[7] Song J.W., Hong S.-B., Lim C.-M., Koh Y., Kim D.S. (2011). Acute exacerbation of idiopathic pulmonary fibrosis: Incidence, risk factors and outcome. Eur. Respir. J..

[8] Martinez F.J., Collard H.R., Pardo A., Raghu G., Richeldi L., Selman M., Swigris J.J., Taniguchi H., Wells A.U. (2017). Idiopathic pulmonary fibrosis. Nat. Rev. Dis. Primers.

[9] Mekov E., Miravitlles M., Petkov R. (2020). Artificial intelligence and machine learning in respiratory medicine. Expert Rev. Respir. Med..

[10] Walsh S.L.F., Humphries S.M., Wells A.U., Brown K.K. (2020). Imaging research in fibrotic lung disease; applying deep learning to unsolved problems. Lancet Respir. Med..

[11] Walsh S.L.F., Calandriello L., Silva M., Sverzellati N. (2018). Deep learning for classifying fibrotic lung disease on high-resolution computed tomography: A case-cohort study. Lancet Respir. Med..

[12] Schwartz D., Helmers R.A., Galvin J.R., Van Fossen D.S., Frees K.L., Dayton C.S., Burmeister L.F., Hunninghake G.W. (1994). Determinants of survival in idiopathic pulmonary fibrosis. Am. J. Respir. Crit. Care Med..

[13] Raghu G., Weycker D., Edelsberg J., Bradford W.Z., Oster G. (2006). Incidence and prevalence of idiopathic pulmonary fibrosis. Am. J. Respir. Crit. Care Med..

[14] Olson A.L., Swigris J.J. (2012). Idiopathic pulmonary fibrosis: Diagnosis and epidemiology. Clin. Chest Med..

[15] King T.E., Tooze J.A., Schwarz M.I., Brown K.R., Cherniack R.M. (2001). Predicting survival in idiopathic pulmonary fibrosis: Scoring system and survival model. Am. J. Respir. Crit. Care Med..

[16] Ryerson C.J., Hartman T., Elicker B.M., Ley B., Lee J.S., Abbritti M., Jones K.D., King T.E., Ryu J., Collard H.R. (2013). Clinical features and outcomes in combined pulmonary fibrosis and emphysema in idiopathic pulmonary fibrosis. Chest.

[17] Fell C.D., Martinez F.J., Liu L.X., Murray S., Han M.K., Kazerooni E.A., Gross B.H., Myers J., Travis W.D., Colby T.V. (2010). Clinical predictors of a diagnosis of idiopathic pulmonary fibrosis. Am. J. Respir. Crit. Care Med..

[18] Shi Y., Wong W.K., Goldin J.G., Brown M.S., Kim G.H.J. (2019). Prediction of progression in idiopathic pulmonary fibrosis using CT scans at baseline: A quantum particle swarm optimization-Random Forest approach. Artif. Intell. Med..

[19] Christe A., Peters A.A., Drakopoulos D., Heverhagen J., Geiser T., Stathopoulou T., Christodoulidis S., Anthimopoulos M., Mougiakakou S.G., Ebner L. (2019). Computer-aided diagnosis of pulmonary fibrosis using deep learning and CT images. Investig. Radiol..

[20] Hussain A., Choi H.-E., Kim H.-J., Aich S., Saqlain M., Kim H.-C. (2021). Forecast the Exacerbation in Patients of Chronic Obstructive Pulmonary Disease with Clinical Indicators Using Machine Learning Techniques. Diagnostics.

[21] Park S.C., Tan J., Wang X., Lederman D., Leader J.K., Kim S.H., Zheng B. (2011). Computer-aided detection of early interstitial lung diseases using low-dose CT images. Phys. Med. Biol..

[22] Zelaya C.V.G. Towards explaining the effects of data preprocessing on machine learning. Proceedings of the 2019 IEEE 35th International Conference on Data Engineering (ICDE).

[23] García S., Luengo J., Herrera F. (2015). Data Preprocessing in Data Mining.

[24] Newgard C.D., Lewis R.J. (2015). Missing data: How to best account for what is not known. JAMA.

[25] Zhang S. (2012). Nearest neighbor selection for iteratively kNN imputation. J. Syst. Softw..

[26] Ghaemi M., Feizi-Derakhshi M.-R. (2016). Feature selection using forest optimization algorithm. Pattern Recognit..

[27] Han W., Huang Z., Li S., Jia Y. (2019). Distribution-sensitive unbalanced data oversampling method for medical diagnosis. J. Med Syst..

[28] Chawla N.V., Bowyer K.W., Hall L.O., Kegelmeyer W.P. (2002). SMOTE: Synthetic minority over-sampling technique. J. Artif. Intell. Res..

[29] Chandrashekar G., Sahin F. (2014). A survey on feature selection methods. Comput. Electr. Eng..

[30] Ren Y., Zhang L., Suganthan P. (2016). Ensemble classification and regression-recent developments, applications and future directions. IEEE Comput. Intell. Mag..

